# Supplementary material for: Treatment of Plasmodium falciparum merozoites with the protease inhibitor E64 and mechanical filtration increases their susceptibility to complement activation
Source: PLoS One. 2020 Aug 21;15(8):e0237786. doi: 10.1371/journal.pone.0237786 (PMC7442247; doi:10.1371/journal.pone.0237786)

**S1 Fig Gating Strategy**: A) Sample of merozoites filtered through a 1.2 um filter and incubated in FS for 15 min was acquired using logarithmic amplification on side scatter vs. forward scatter plot. B) Single merozoites were gated on the basis of low side scatter and positive Hoechst staining as well as on positive staining with Alexa 488-conjugated MAb5.2 (C). D) Dot plot of measurement of merozoite membrane integrity. E) Histogram of merozoites from panel D. F) After addition of FITC-labeled anti-C3b or anti-C5b9 antibodies, we measured the percent FITC positive population. EXP-18-FJ5475

B

A


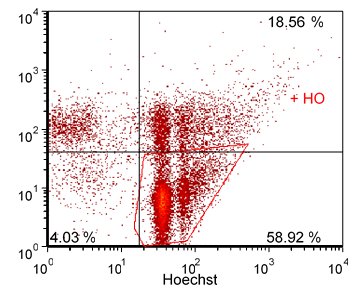

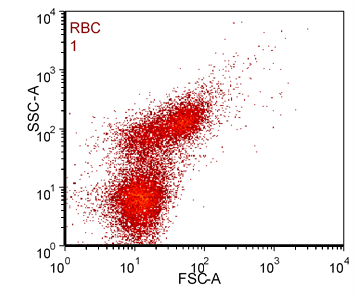


Live-dead Reagent

D

C

MAb5.2 Alexa 488 or IgG2b Alexa 488


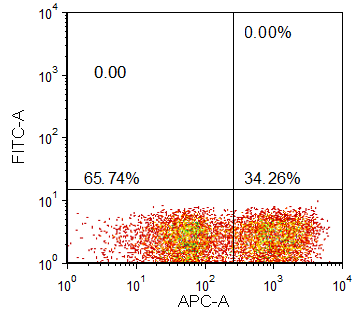

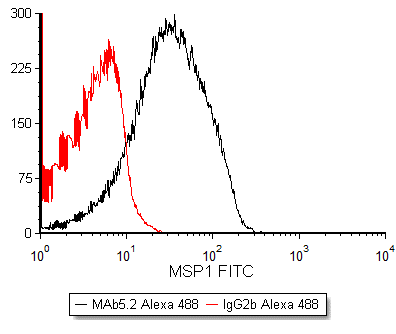


Anti-C3b or Anti-C5b9 FITC

F

E


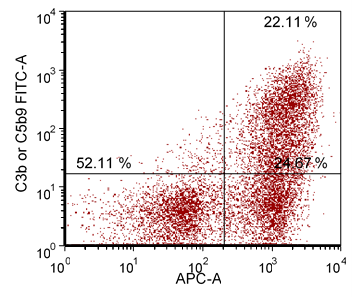


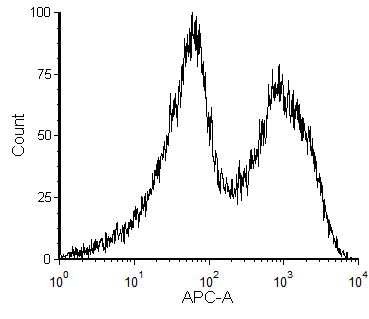

Supplement: S1 Fig — A) Sample of merozoites filtered through a 1.2 um filter and incubated in FS for 15 min was acquired using logarithmic amplification on side scatter vs. forward scatter plot. B) Single merozoites were gated on the basis of low side scatter and positive Hoechst staining as well as on positive staining with Alexa 488-conjugated MAb5.2 (C). D) Dot plot of measurement of merozoite membrane integrity. E) Histogram of merozoites from panel D. F) After addition of FITC-labeled anti-C3b or anti-C5b9 antibodies, we measured the percent FITC positive population. EXP-18-FJ5475. (DOCX) [file pone.0237786.s001.docx]
