# Supplementary material for: Treatment of Plasmodium falciparum merozoites with the protease inhibitor E64 and mechanical filtration increases their susceptibility to complement activation
Source: PLoS One. 2020 Aug 21;15(8):e0237786. doi: 10.1371/journal.pone.0237786 (PMC7442247; doi:10.1371/journal.pone.0237786)
Supplement: S2 Fig — A) Effect of filtration on membrane integrity of E64-treated merozoites in FS. B) Effect of filtration on membrane integrity of E64-untreated merozoites in FS. C) Effect of filtration on membrane integrity of E64-treated merozoites in HIS. D) Effect of filtration on membrane integrity of E64-untreated merozoites in HIS. *P < 0.05 for the comparison between unfiltered and 1.2 μm filtered merozoites (Panels A and B), and for the comparison between unfiltered and filtered merozoites (Panels C and D). Error bars represent standard errors of the mean. (DOCX) [file pone.0237786.s002.docx]

**S2 Fig** **Effect of filtration on merozoite membrane integrity.** A) Effect of filtration on membrane integrity of E64-treated merozoites in FS. B) Effect of filtration on membrane integrity of E64-untreated merozoites in FS. C) Effect of filtration on membrane integrity of E64-treated merozoites in HIS. D) Effect of filtration on membrane integrity of E64-untreated merozoites in HIS. *P < 0.05 for the comparison between unfiltered and 1.2 µm filtered merozoites (Panels A and B), and for the comparison between unfiltered and filtered merozoites (Panels C and D). Error bars represent standard errors of the mean.
