# Supplementary material for: Treatment of Plasmodium falciparum merozoites with the protease inhibitor E64 and mechanical filtration increases their susceptibility to complement activation
Source: PLoS One. 2020 Aug 21;15(8):e0237786. doi: 10.1371/journal.pone.0237786 (PMC7442247; doi:10.1371/journal.pone.0237786)
Supplement: S3 Fig — A) Effect of E64 treatment on membrane integrity of unfiltered merozoites in HIS. B) Effect of E64 treatment on membrane integrity of filtered merozoites in HIS. C) Effect of E64 treatment on membrane integrity of unfiltered merozoites in FS. D) Effect of E64 treatment on membrane integrity of filtered merozoites in FS. *P < 0.05 for the comparison between E64-treated and untreated merozoites (Panel C) and the comparison between 1.2 μm filtered merozoites (Panel D). Error bars represent standard errors of the mean. (DOCX) [file pone.0237786.s003.docx]

**S3 Fig Effect of E64 treatment on merozoite membrane integrity.** A) Effect of E64 treatment on membrane integrity of unfiltered merozoites in HIS. B) Effect of E64 treatment on membrane integrity of filtered merozoites in HIS. C) Effect of E64 treatment on membrane integrity of unfiltered merozoites in FS. D) Effect of E64 treatment on membrane integrity of filtered merozoites in FS. *P < 0.05 for the comparison between E64-treated and untreated merozoites (Panel C) and the comparison between 1.2 µm filtered merozoites (Panel D). Error bars represent standard errors of the mean.
