# Supplementary material for: Treatment of Plasmodium falciparum merozoites with the protease inhibitor E64 and mechanical filtration increases their susceptibility to complement activation
Source: PLoS One. 2020 Aug 21;15(8):e0237786. doi: 10.1371/journal.pone.0237786 (PMC7442247; doi:10.1371/journal.pone.0237786)
Supplement: S4 Fig — A) Effect of serum and filtration on the membrane integrity of E64-untreated merozoites in the presence of IgG2b. B) Effect of serum and filtration on the membrane integrity of E64-untreated merozoites in the presence of MAb5.2. C) Effect of serum and filtration on the membrane integrity of E64-treated merozoites in the presence of IgG2b. D) Effect of serum and filtration on the integrity of E64-treated merozoites in the presence of MAb5.2. Error bars represent standard errors of the mean. (DOCX) [file pone.0237786.s004.docx]

**S4 Fig Effect of serum and filtration on the membrane integrity of merozoites in IgG2b or MAb5.2.** A) Effect of serum and filtration on the membrane integrity of E64-untreated merozoites in the presence of IgG2b. B) Effect of serum and filtration on the membrane integrity of E64-untreated merozoites in the presence of MAb5.2. C) Effect of serum and filtration on the membrane integrity of E64-treated merozoites in the presence of IgG2b. D) Effect of serum and filtration on the integrity of E64-treated merozoites in the presence of MAb5.2. Error bars represent standard errors of the mean.
