# Supplementary material for: Treatment of Plasmodium falciparum merozoites with the protease inhibitor E64 and mechanical filtration increases their susceptibility to complement activation
Source: PLoS One. 2020 Aug 21;15(8):e0237786. doi: 10.1371/journal.pone.0237786 (PMC7442247; doi:10.1371/journal.pone.0237786)
Supplement: S5 Fig — A) Effect of E64 and serum on the membrane integrity of unfiltered merozoites in the presence of IgG2b. B) Effect of E64 and serum on the membrane integrity of unfiltered merozoites in the presence of MAb5.2. C) Effect of E64 and serum on the membrane integrity of filtered merozoites in the presence of IgG2b. D) Effect of E64 and serum on the membrane integrity of filtered merozoites in the presence of MAb5.2. Error bars represent standard errors of the mean. (DOCX) [file pone.0237786.s005.docx]

**S5 Fig Effect of E64 and serum on the integrity of merozoites in the presence of IgG2b or MAb5.2.** A) Effect of E64 and serum on the membrane integrity of unfiltered merozoites in the presence of IgG2b. B) Effect of E64 and serum on the membrane integrity of unfiltered merozoites in the presence of MAb5.2. C) Effect of E64 and serum on the membrane integrity of filtered merozoites in the presence of IgG2b. D) Effect of E64 and serum on the membrane integrity of filtered merozoites in the presence of MAb5.2. Error bars represent standard errors of the mean.
