# Supplementary material for: Treatment of Plasmodium falciparum merozoites with the protease inhibitor E64 and mechanical filtration increases their susceptibility to complement activation
Source: PLoS One. 2020 Aug 21;15(8):e0237786. doi: 10.1371/journal.pone.0237786 (PMC7442247; doi:10.1371/journal.pone.0237786)
Supplement: S7 Fig — Following Percoll enrichment schizonts were incubated overnight in complete media containing HIS with or without 10 uM E64. After removal of E64, merozoites were allowed to egress. Panel B shows increased proportion of clumped merozoites compared to panel A, which was confirmed by microscopic examination. EXP-18-FJ5475. (DOCX) [file pone.0237786.s007.docx]

**S7 Fig Effect of Filtration and E64 Treatment on Membrane Integrity of Low and High Hoechst Merozoites**. Low Hoechst merozoites tre. B) High Hoechst Merozoites. Low Hoechst merozoites show less membrane damage than high Hoechst merozoites. In both groups, E64-treated merozoites filtered with the 1.2 um filter show less membrane damage at baseline but they rapidly deteriorate after addition of fresh serum (FS) and by time 0 there is not significant difference. *P<0.01 for the comparison between 1.2um-filtered merozoites and unfiltered merozoites. **P<0.05 for the comparison of 1.2um-filtered merozoites with the other two groups. Error bars represent standard errors.
