# Supplementary material for: Treatment of Plasmodium falciparum merozoites with the protease inhibitor E64 and mechanical filtration increases their susceptibility to complement activation
Source: PLoS One. 2020 Aug 21;15(8):e0237786. doi: 10.1371/journal.pone.0237786 (PMC7442247; doi:10.1371/journal.pone.0237786)
Supplement: S8 Fig — Free merozoites purified by filtration through a 1.2 um filtered were incubated in complete media containing 10% HIS and RBCs at 0.5% hematocrit for a few seconds (A) or 15 minutes (B). The RBCs were washed once and incubated in complete medium overnight. The following day a sample was obtained for flow cytometry and stained with Hoechst 33342. The apparent percent IRBCs increased from 2.5% to 3.6%. However, microscopic examination showed that the vast majority of these invasive forms were merozoites attached to the surface of RBCs. EXP-18-FJ5497. (DOCX) [file pone.0237786.s008.docx]

**S8 Fig Effect of Filtration and E64 Treatment on the Deposition of C3b and C5b9 on Low and High Hoechst Membrane-damaged Merozoites**: A) Effect of filtration and E64 treatment on the deposition of C3b on low Hoechst merozoites. B) Effect of filtration and E64 treatment on the deposition of C3b on high Hoechst merozoites. C) Effect of filtration and E64 treatment on the deposition of C5b9 on low Hoechst merozoites. D) Effect of filtration and E64 treatment on the deposition of C5b9 on high Hoechst merozoites. *P<0.001, **P<0.01, and ***P<0.05 for the comparison of 1.2um-filtered merozoites with the other two groups.
