# Supplementary material for: Treatment of Plasmodium falciparum merozoites with the protease inhibitor E64 and mechanical filtration increases their susceptibility to complement activation
Source: PLoS One. 2020 Aug 21;15(8):e0237786. doi: 10.1371/journal.pone.0237786 (PMC7442247; doi:10.1371/journal.pone.0237786)
Supplement: S9 Fig — A) Effect of filtration on event rate of E64-untreated merozoites in FS. B) Effect of filtration on event rate of E64-untreated merozoites in HIS. C) Effect of filtration on event rate of E64-treated merozoites in FS. D) Effect of filtration on event rate of E64-treated merozoites in HIS. (DOCX) [file pone.0237786.s009.docx]

**S9 Fig Effect of E64 and Filtration on Merozoite Event Rate**. A) Effect of filtration on event rate of E64-untreated merozoites in FS. B) Effect of filtration on event rate of E64-untreated merozoites in HIS. C) Effect of filtration on event rate of E64-treated merozoites in FS. D) Effect of filtration on event rate of E64-treated merozoites in HIS.
