# Supplementary material for: Treatment of Plasmodium falciparum merozoites with the protease inhibitor E64 and mechanical filtration increases their susceptibility to complement activation
Source: PLoS One. 2020 Aug 21;15(8):e0237786. doi: 10.1371/journal.pone.0237786 (PMC7442247; doi:10.1371/journal.pone.0237786)
Supplement: S10 Fig — Following Percoll enrichment schizonts were incubated overnight in complete media containing HIS with or without 10 uM E64. After removal of E64, merozoites were allowed to egress. Panel B shows increased proportion of clumped merozoites compared to panel A, which was confirmed by microscopic examination. (DOCX) [file pone.0237786.s010.docx]

**S10 Fig E64 Causes Clumping of Merozoites**. Following Percoll enrichment schizonts were incubated overnight in complete media containing HIS with or without 10 uM E64. After removal of E64, merozoites were allowed to egress. Panel B shows increased proportion of clumped merozoites compared to panel A, which was confirmed by microscopic examination. EXP-18-FJ5475

Infected RBCs and Clumped Merozoites

1. Unfiltered HIS No E64

B) Unfiltered HIS E64-treated


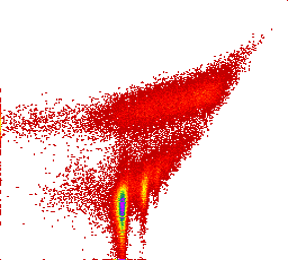


BV421-A

SSC-A

10

0

10

1

10

2

10

3

10

4

10

0

10

1

10

2

10

3

10

4

61.43

%

1.52

%

34.36

%


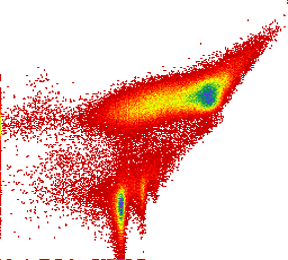


BV421-A

SSC-A

10

0

10

1

10

2

10

3

10

4

10

0

10

1

10

2

10

3

10

4

23.26

%

1.88

%

72.65

%

Single Merozoites
