# Supplementary material for: Treatment of Plasmodium falciparum merozoites with the protease inhibitor E64 and mechanical filtration increases their susceptibility to complement activation
Source: PLoS One. 2020 Aug 21;15(8):e0237786. doi: 10.1371/journal.pone.0237786 (PMC7442247; doi:10.1371/journal.pone.0237786)
Supplement: S11 Fig — Free merozoites purified by filtration through a 1.2 um filtered were incubated in complete media containing 10% HIS and RBCs at 0.5% hematocrit for a few seconds (A) or 15 minutes (B). The RBCs were washed once and incubated in complete medium overnight. The following day a sample was obtained for flow cytometry and stained with Hoechst 33342. The apparent percent IRBCs increased from 2.5% to 3.6%. However, microscopic examination showed that the vast majority of these invasive forms were merozoites attached to the surface of RBCs. (DOCX) [file pone.0237786.s011.docx]

**S11 Fig Measurement of Invasion**. Free merozoites purified by filtration through a 1.2 um filtered were incubated in complete media containing 10% HIS and RBCs at 0.5% hematocrit for a few seconds (A) or 15 minutes (B). The RBCs were washed once and incubated in complete medium overnight. The following day a sample was obtained for flow cytometry and stained with Hoechst 33342. The apparent percent IRBCs increased from 2.5% to 3.6%. However, microscopic examination showed that the vast majority of these invasive forms were merozoites attached to the surface of RBCs. EXP-18-FJ5497

B

A
