# Supplementary material for: Treatment of Plasmodium falciparum merozoites with the protease inhibitor E64 and mechanical filtration increases their susceptibility to complement activation
Source: PLoS One. 2020 Aug 21;15(8):e0237786. doi: 10.1371/journal.pone.0237786 (PMC7442247; doi:10.1371/journal.pone.0237786)
Supplement: Attachment — Submitted filename: Response to Reviewers 6-2-20.docx [file pone.0237786.s012.docx]

Response to reviewers 6-2-20.

"In Fig S1B and Fig S7, the gated population as single merozoites looks containing single and doublet merozoites. Mixed population may affect all subsequent data. This gated single merozoites population region indicated by authors could be divided into two, for example, the regions could be separated by 50 and 80 of Hoechst value in Fig S1B and Fig S7, respectively. To prove the result, authors need to reanalyze the data with single merozoites as above and the all of plot data set should be included in supplemental figure."

R: I thank the reviewers for their additional comments above. We have carried out additional data analysis comparing the low and high Hoechst populations of merozoites. The data for this analysis are now located in Supplemental Figs S6-8. Essentially, we divided merozoites into low and high Hoechst stained merozoites and compared their membrane integrity and their deposition of C3b and C5b9. The major difference between the two was that high Hoechst merozoites have a higher proportion of membrane-damaged merozoites than low Hoechst merozoites. This suggests that very likely their differential staining of Hoechst is related to their difference in membrane integrity. We found that in both populations the use of the combination of E64 and filtration led to increased deposition of C3b and C5b9. However, the high Hoechst merozoites always showed greater deposition of these complement effector molecules. Therefore, our original analysis using the whole population is accurate.

In addition to the above, we have made minor corrections to the manuscript to enhance clarity.
